# A195 IL-13 DRIVES CROHN’S DISEASE ASSOCIATED-INTESTINAL FIBROSIS IN SHIP DEFICIENT MICE

**DOI:** 10.1093/jcag/gwad061.195

**Published:** 2024-02-14

**Authors:** K Safari, S C Menzies, L M Sly

**Affiliations:** BC Children's Hospital Research Institute, Vancouver, BC, Canada; BC Children's Hospital Research Institute, Vancouver, BC, Canada; BC Children's Hospital Research Institute, Vancouver, BC, Canada

## Abstract

**Background:**

Crohn’s disease (CD) is an immune-mediated disease characterized by chronic, relapsing and remitting, or progressive inflammation of the digestive tract. One in 3 people with CD will develop intestinal fibrosis requiring surgery within 10 years of diagnosis. Biological therapy is effective at reducing inflammation in CD and early and aggressive treatment with biologics will reduce the incidence of fibrosis. However, in children, for whom a step-up approach to therapy is used, and populations that are refractory to biological therapy; fibrosis remains a serious concern. Treatments are urgently needed as currently there are no treatments that target intestinal fibrosis directly in CD.

Mice deficient in the Src homology 2 domain-containing inositolpolyphosphate 5’-phosphatase (SHIP-/-) develop spontaneous CD-like ileal inflammation and fibrosis. Fibrosis is dependent on PI3Kp110δ activity. IL-4 and IL-13 lead to downstream activation of PI3Kp110δ. Recent work in our lab has shown through pharmacological inhibition that the development of fibrosis in SHIP-/- mice is dependenet on IL-13 signaling, but not IL-4.

**Aims:**

Aim 1: To determine whether genetic ablation of IL-13 prevents ileal fibrosis in SHIP-/- mice

Aim 2: To determine the cellular source of IL-13 in the distal ileum of SHIP-/- mice

Aim 3: To determine the cellular source of IL-13 in the distal ileum of individuals with CD

**Methods:**

SHIP-/- mice were crossed with IL-13 deficient mice (IL13-/-) to generate SHIP+/+IL13+/+, SHIP+/+IL13-/-, SHIP-/-IL13+/+, and SHIP-/-IL13-/- mice. Measurements of ileal fibrosis included gross and histopathology, muscle thickening, and collagen accumulation (Masson’s trichrome staining for fibrosis). Single cell RNA sequencing (snRNA-seq) was performed on the distal ileum of SHIP-/- mice to determine the cellular source of IL-13. Previously published scRNA-seq data were analyzed to determine the cellular source of IL-13 in individuals with CD.

**Results:**

Genetic ablation of IL-13 in SHIP-/- mice (SHIP-/-IL13-/-) reduced the development of ileal fibrosis. Analysis of single cell RNA sequencing data (scRNA seq) has revealed mast cells as the cellular source of IL-13 in the distal ileum of people with CD.

**Conclusions:**

Mast cell derived IL-13 may be driving the development of intestinal fibrosis in CD. Our data suggest that an antibody-based biologic targeting IL-13 may be an effective strategy to prevent fibrosis in people with Crohn’s Disease.

**Funding Agencies:**

CAG, CIHR

